# Corporate Governance and Workplace Mental Health Practices: The Mediating Role of Structured Occupational Safety and Health Engagement

**DOI:** 10.1016/j.shaw.2025.12.003

**Published:** 2026-01-03

**Authors:** Ro-Ting Lin, Lung-Chang Chien, Chieh-Wen Chang, Yu-Chi Liao, Tomohisa Nagata

**Affiliations:** 1 Department of Occupational Safety and Health, College of Public Health, China Medical University, Taichung, Taiwan; 2 Department of Epidemiology and Biostatistics, School of Public Health, University of Nevada Las Vegas, Las Vegas, NV, United States; 3 Department of Psychology, College of Medical and Health Science, Asia University, Taichung, Taiwan; 4 Department of Occupational Health Practice and Management, Institute of Industrial Ecological Sciences, University of Occupational and Environmental Health, Fukuoka, Japan

**Keywords:** Corporate governance, Mediation analysis, Occupational safety and health, Sustainability reporting, Workplace mental health

## Abstract

**Background:**

This study examines whether stronger corporate governance is associated with workplace mental health practices and whether this relationship is mediated by a structured sequence of occupational safety and health (OSH) engagement: recognition, goal-setting, and implementation.

**Methods:**

We analyzed 134 listed companies in Taiwan that published sustainability reports and received corporate governance evaluations between 2014 and 2023. Governance scores—based on shareholder rights, board functioning, transparency, and sustainability—were dichotomized into high vs. low categories. Workplace mental health practices were measured using 24 binary indicators across planning, provision, and reporting. OSH engagement was conceptualized in three stages based on Global Reporting Initiative 403 guidelines, with composite indicators derived via grouped weighted quantile sum regression. Associations were analyzed using generalized linear mixed-effects and serial mediation models.

**Results:**

Compared to companies ranked low in corporate governance, those ranked high had a higher likelihood of adopting mental health practices. OSH goal-setting and implementation showed significant positive associations, whereas recognition did not. Serial mediation analysis showed that 83% of the total effect of corporate governance on mental health practices was direct, while 17% was mediated through structured OSH engagement, primarily via implementation. Recognition showed a non-significant effect but initiated significant sequential pathways via goal-setting and implementation. Goal-setting functioned as a bridge within these chains.

**Conclusion:**

Corporate governance plays a central role in advancing workplace mental health practices. While most effects are direct, structured OSH engagement helps translate governance priorities into sustained organizational actions that embed mental health into routine practice.

## 1 Introduction

Mental health is a growing concern among working-age adults [1], with around 15% having a diagnosable mental disorder at any given time [2], highlighting the workplace as a critical setting for intervention. Psychosocial risk factors—such as excessive working hours, adverse psychosocial work conditions, workplace violence, and harassment—have been linked to depression and suicide [[3], [4], [5], [6], [7], [8]].

In response to increased awareness of psychosocial risks, international organizations, such as the United Nations, the World Health Organization, and the International Labour Organization, have issued frameworks emphasizing mental well-being in the workplace [2,9,10]. National governments, such as those in Japan, the Republic of Korea, and Taiwan, have enacted legislation to mitigate these risks [[11], [12], [13]]. For example, Taiwan's Occupational Safety and Health Act obligates employers to address overwork, workplace violence, and related psychosocial hazards [13]. Collectively, these developments have positioned mental health as a core component of occupational safety and health (OSH), reinforced by standards such as ISO 45003 and the Global Reporting Initiative (GRI) 403, which call for the integration of psychosocial risk management into OSH systems and disclosure through sustainability reporting [14,15].

However, the adoption and implementation of mental health practices vary considerably across organizations, often shaped by internal governance structures. Corporate governance plays a pivotal role in how companies prioritize, institutionalize, and disclose OSH and mental health practices [[16], [17], [18]]. Through board-level oversight, strategic planning, and internal accountability mechanisms, governance influences both operational execution and transparency [19,20]. For example, during the COVID-19 pandemic, Taiwanese-listed companies with stronger governance were more likely to implement online mental health programs [21]. These relationships align with safety management system principles, which emphasize leadership commitment, managerial behaviors, and formalized structures as drivers of organizational responses to OSH challenges [22,23]. Evidence from safety leadership research likewise shows that visible senior management support, reinforced through policies and structured processes, shapes safety climate and behavior [22,23]. Together, these mechanisms explain how governance can direct the prioritization and implementation of workplace mental health practices [24,25]. Governance also shapes sustainability reporting behaviors [26,27], influencing how companies disclose OSH and mental health practices under frameworks like GRI 403.

Organizational responses to mental health often evolve through distinct stages—beginning with recognition of its importance, followed by the establishment of formal goals, and culminating in the implementation of concrete measures [14,15]. Building on this progression, we define structured OSH engagement as a three-stage process: (1) recognition, referring to whether OSH is explicitly identified as an organizational priority; (2) goal-setting, involving the establishment of strategic objectives and formal targets; and (3) implementation, which entails executing systems, training, and operational practices [28]. These stages reflect increasing levels of organizational commitment and capacity to address psychosocial risks.

While the mental health risks associated with psychosocial hazards are well documented [[3], [4], [5], [6], [7], [8]], how companies translate governance structures into concrete mental health practices and OSH engagement remains underexplored. Prior studies focus on individual or general OSH factors rather than governance as an upstream determinant [[3], [4], [5], [6], [7], [8],^22^,^23^], despite its role in leadership commitment and institutionalized OSH responsibilities. In a context of growing transparency demands, the present study investigates whether stronger corporate governance is associated with the adoption of workplace mental health practices and whether this relationship is mediated by a structured sequence of OSH engagement: recognition, goal-setting, and implementation. Specifically, this study aims to: (1) examine the association between governance strength and workplace mental health practices; and (2) assess whether OSH recognition, goal-setting, and implementation mediate this relationship.

## 2 Materials and methods

### 2.1 Study design

This longitudinal observational study examined the association between corporate governance, structured OSH engagement, and workplace mental health practices among publicly listed companies in Taiwan. The main outcome was the annual extent of workplace mental health practice adoption, based on disclosures in corporate sustainability reports. Corporate governance and OSH engagement were treated as predictors. The dataset consisted of repeated company-year observations, forming a balanced 10-year panel structure suitable for longitudinal analysis.

### 2.2 Setting

The study focused on companies listed on the Taiwan Stock Exchange and the Taipei Exchange. These companies are subject to annual corporate governance evaluations and sustainability disclosure requirements, which may be either mandatory or voluntary depending on paid-in capital and ownership structure [29,30]. The analysis covered corporate activities from 2014 to 2023. Due to the typical one-year lag in the release of sustainability reports, data collection spanned the period from 2015 to 2025. The baseline year, 2014, was chosen because it marked two critical developments: the enactment of Taiwan's revised Occupational Safety and Health Act and the launch of the Corporate Governance Evaluation System [13,31,32]. This setting allows governance and reporting obligations to shape OSH and mental health disclosures over time.

### 2.3 Study size

We initially identified 242 companies with complete governance evaluation records from 2014 to 2023 [32] and 279 companies that published sustainability reports in all 10 years on the Market Observation Post System (MOPS) [33]. After merging datasets and retaining only companies with full 10-year coverage in both sources, the final analytical sample included 134 companies (1,340 company-year observations). Companies with missing governance evaluations or incomplete sustainability reports in any year were excluded to ensure consistent longitudinal comparisons. Of these, 109 (81%) companies were listed on the Taiwan Stock Exchange, and 25 (19%) on the Taipei Exchange in 2014, with their listing status remaining stable through 2023. Mandatory disclosure applied to 85 companies (63%) at baseline and increased to 110 (82%) by 2023 as regulations expanded. The sample reflects all companies with complete 10-year data, so the size was determined by data availability. We acknowledge that excluding companies with intermittent reporting may introduce selection bias.

### 2.4 Variables and data sources

The study focused on four variable domains: (1) corporate governance rankings, (2) OSH engagement indicators, (3) workplace mental health practices, and (4) company-level characteristics. All data were derived from publicly available secondary sources.

Corporate governance was assessed using rankings from the Corporate Governance Evaluation System, maintained by the Taiwan Stock Exchange and the Taipei Exchange [31,32]. The evaluation covers four main dimensions: shareholder rights, board structure and functioning, information transparency, and sustainable development [31,32]. Companies were classified into high or low governance groups based on their annual percentile rankings. Companies ranked in the top 5% were designated as high governance; all others were categorized as low governance (Supplementary Section 1).

OSH engagement was assessed using 23 binary indicators extracted from annual sustainability reports and grouped into three stages: recognition (7 indicators), goal-setting (6), and implementation (10), developed based on GRI 403 standards, ISO 45001 guidelines, and prior research [14,[34], [35], [36], [37], [38]]. Reports were manually reviewed, and indicators were coded as 1 (disclosed) or 0 (not disclosed) based on standardized definitions (Supplementary Section 2). For OSH engagement and mental health indicators, missing values were treated as non-disclosure in sustainability reports and coded as 0 per the predefined coding protocol. Stage-specific scores were aggregated at the company-year level. Binary coding was selected to maximize inter-year comparability across heterogeneous narrative disclosures, which often lack standardized quantitative detail.

Workplace mental health practices were assessed using 24 binary indicators across three categories: planning (two indicators), provision (15 indicators), and reporting (seven indicators). These indicators were developed by integrating GRI 403 reporting principles, ISO 45003 guidance, and prior research [14,15,37]. While GRI 403 does not explicitly reference mental health, its structured approach to occupational health and safety reporting was adapted to assess mental health-related disclosures, with ISO 45003 providing specific guidance on psychosocial risks and mental well-being. The planning category captured whether companies disclosed formal commitments to address psychosocial risks (e.g., abnormal workloads, workplace violence). The provision category reflected available systems and support programs, such as psychological counseling services, employee assistance programs, and working time management. The reporting category assessed whether organizations disclosed participation rates or outcome statistics related to mental health initiatives. Indicators were coded as 1 if disclosed and 0 if not, and annual scores were summed to create a workplace mental health index (Supplementary Section 3). Each company-year observation was manually coded by two trained coders following calibration rounds to ensure consistent interpretation of indicator definitions, with discrepancies resolved through consensus where applicable, and a few cases verified via keyword searches (Adobe Acrobat Pro).

All OSH and mental health data were extracted from sustainability reports, including corporate social responsibility, environmental, social, and governance (ESG) disclosures, and other related publications available on MOPS [33]. These reports were reviewed by trained coders using a standardized coding protocol developed in prior research [[36], [37], [38]].

Company characteristics included market type (defined as listing on the Taiwan Stock Exchange or the Taipei Exchange) and industry category (classified as manufacturing, wholesale and retail trade, finance and insurance, or other sectors), based on the Executive Yuan's classification system and MOPS documentation [33,39] (Supplementary Section 4).

### 2.5 Bias management

To address reporting bias, we conducted a sensitivity analysis comparing mandatory and voluntary disclosure regimes. Voluntary reporters may selectively disclose favorable information, whereas mandatory reporters may comply superficially, making some OSH and mental health disclosures symbolic rather than reflective of actual implementation. Selection bias was minimized by including only companies with complete longitudinal data. Observer bias was reduced through a standardized coding protocol, double coding or keyword verification (Adobe Acrobat Pro), and consensus-based resolution of discrepancies. Inter-rater reliability was assessed using Cohen's Kappa, observed agreement, and prevalence-adjusted bias-adjusted Kappa (PABAK), with mean and standard deviation reported across items. Internal consistency was evaluated using Cronbach's alpha based on tetrachoric correlations, with no item reversal, as all indicators reflected the same directional construct.

### 2.6 Statistical analysis

We employed a multi-step approach to examine how corporate governance relates to workplace mental health practices, considering the mediating role of structured OSH engagement (recognition, goal-setting, and implementation). Statistical details are provided in Supplementary Section 5.

Briefly, we first used grouped weighted quantile sum (GWQS) regression to construct composite indices for each OSH stage, addressing multicollinearity among binary indicators and generating annual weighted scores. Second, we applied generalized linear mixed-effects models (GLMMs) to estimate longitudinal associations from 2014 to 2023. The outcome was the annual count of disclosed mental health practices. Independent variables included governance group, the three GWQS indices (standardized by interquartile range), year, market type, and industry category. Company-level clustering was modeled using a first-order autoregressive structure. Incidence rate ratios (IRRs) were used to interpret effect sizes. Finally, we conducted serial mediation analysis to assess whether OSH engagement stages mediated the relationship between governance and mental health practices. Governance served as the predictor; the three OSH engagement stages were modeled as sequential mediators; and the mental health score was the outcome. Total, direct, and indirect effects were estimated using 5,000 bootstrap samples.

All analyses were conducted using R (version 4.3.3) in RStudio (version 2024.12.1, Build 563). Statistical significance was set at *p* < 0.05.

## 3 Results

**Fig. 1:**
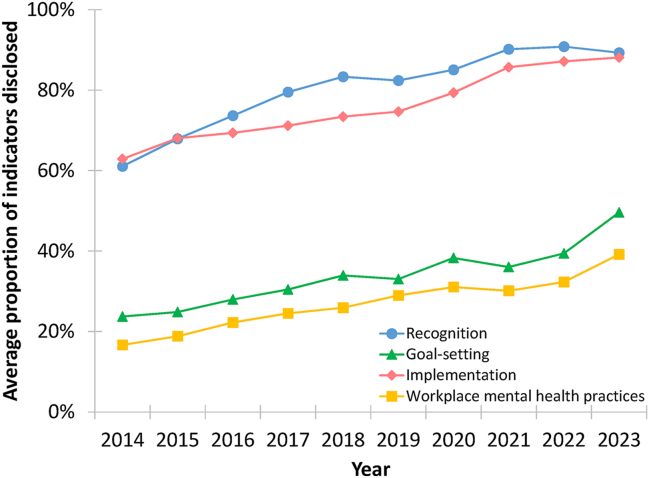
Trends in the proportion of disclosed indicators across occupational safety and health engagement dimensions and workplace mental health practices, 2014–2023.

Fig. 1 shows the annual average proportion of disclosed indicators from 2014 to 2023 across the three OSH engagement stages and workplace mental health practices. Recognition and implementation consistently had high disclosure levels (89% and 88% in 2023), while goal-setting remained low but increased sharply in 2023. Mental health practice disclosure steadily increased from 17% to over 39%. Interrater reliability was high, with 96% agreement, a Cohen's Kappa of 0.84 (SD = 0.14), and a PABAK of 0.92 (SD = 0.07). Both composite indices showed strong internal consistency (Cronbach's alpha = 0.93 for OSH engagement; 0.89 for mental health practices).

Table 1 shows that, consistent with the first study aim, the adoption of workplace mental health practices increased steadily after Taiwan's 2014 reform, with the IRR rising from 1.13 (*p* = 0.001) in 2015 to 2.34 (*p* < 0.001) in 2023, relative to the 2014 baseline. Companies with high corporate governance scores were more likely to adopt mental health practices (IRR = 1.13, *p* < 0.001). Goal-setting (IRR = 1.17) and implementation (IRR = 1.14) dimensions of OSH engagement were significant predictors (*p* < 0.001), while recognition was not (IRR = 1.00, *p* = 0.963). No significant differences were observed by industry sector, but companies listed on the Taiwan Stock Exchange had significantly higher adoption than those on the Taipei Exchange (IRR = 1.33, *p* = 0.006).

**Table 1:** Adjusted associations between corporate governance, IQR-standardized OSH engagement components, and workplace mental health practices based on a generalized linear mixed-effects model

Variable	IRR (95% CI)	*p*
Intercept	2.48 (1.91–3.22)	<0.001
Year: 2015 vs. 2014	1.13 (1.05–1.21)	0.001
Year: 2016 vs. 2014	1.34 (1.24–1.46)	<0.001
Year: 2017 vs. 2014	1.47 (1.33–1.62)	<0.001
Year: 2018 vs. 2014	1.55 (1.40–1.71)	<0.001
Year: 2019 vs. 2014	1.72 (1.55–1.90)	<0.001
Year: 2020 vs. 2014	1.84 (1.66–2.03)	<0.001
Year: 2021 vs. 2014	1.78 (1.61–1.98)	<0.001
Year: 2022 vs. 2014	1.93 (1.71–2.17)	<0.001
Year: 2023 vs. 2014	2.34 (2.10–2.61)	<0.001
Governance: High vs. Low	1.13 (1.06–1.20)	<0.001
OSH Recognition	1.00 (0.94–1.06)	0.963
OSH Goal-setting	1.17 (1.12–1.22)	<0.001
OSH Implementation	1.14 (1.08–1.21)	<0.001
Sector: Manufacturing vs. Reference	0.99 (0.85–1.15)	0.900
Sector: Services vs. Reference	1.01 (0.83–1.22)	0.954
Market: TWSE vs. TPEx	1.33 (1.09–1.62)	0.006

Abbreviations: CI, confidence interval; IQR, interquartile range; IRR, incidence rate ratio; OSH, occupational safety and health; TWSE, Taiwan Stock Exchange; TPEx, Taipei Exchange.

**Note**: OSH engagement stages (recognition, goal-setting, and implementation) were standardized by their IQR prior to analysis. IRRs represent the expected change in the outcome per one IQR increase in each respective variable. The reference group for the industry sector is the Finance and Real Estate sector. “Manufacturing” includes industrial and manufacturing companies; “Services” includes commercial and service-related companies.

Table 2 presents the results of the mediation analysis using a serial mediation model, addressing the second study aim. Corporate governance was positively associated with the adoption of workplace mental health practices, with a total effect of 2.19. Seventeen percent of this effect (0.38) was transmitted indirectly through a structured sequence of OSH engagement across three stages: recognition (M_1_), goal-setting (M_2_), and implementation (M_3_). The remaining 83% was attributable to a significant direct effect (1.81). Among the individual indirect pathways, implementation functioned as the strongest standalone mediator (0.20). Although recognition and goal-setting alone did not yield significant indirect effects (paths 1 and 2, respectively), both played important roles within sequential mediation chains. Recognition consistently served as the entry point for multiple significant pathways (paths 4, 5, and 7), initiating downstream engagement processes. Goal-setting, in turn, acted as a critical bridge between early recognition and later implementation, contributing significantly when embedded within these chains (e.g., path 4 and the full path 7).

**Table 2:** Parameter estimates of total, direct, and indirect effects of corporate governance on workplace mental health practices through structured OSH engagement

Effect	Estimate	(95% CI)
Total effect	2.19	(1.75 to 2.62)
Direct effect (not mediated)	1.81	(1.41 to 2.20)
Total indirect effect (mediated via OSH engagement)	0.38	(0.20 to 0.57)
Indirect effect path 1: via OSH recognition (M_1_)	–0.04	(–0.10 to 0.00)
Indirect effect path 2: via OSH goal-setting (M_2_)	0.06	(–0.04 to 0.17)
Indirect effect path 3: via OSH implementation (M_3_)	0.20	(0.08 to 0.34)
Indirect effect path 4: via M_1_ → M_2_	0.03	(0.01 to 0.05)
Indirect effect path 5: via M_1_ → M_3_	0.11	(0.05 to 0.18)
Indirect effect path 6: via M_2_ → M_3_	0.02	(–0.01 to 0.05)
Indirect effect path 7: via M_1_ → M_2_ → M_3_	0.01	(0.00 to 0.02)

Abbreviations: CI, confidence interval; OSH, occupational safety and health.

Note: Based on PROCESS Model 6 with 5,000 bootstrap samples, adjusted for time (linear and quadratic terms), industry, and market status. OSH engagement stages—recognition (M_1_), goal-setting (M_2_), and implementation (M_3_)—were standardized by their interquartile range (IQR). Indirect effects are interpreted per IQR increase in the respective mediator. Effects are considered significant if the 95% confidence interval did not include zero.

**Fig. 2:**
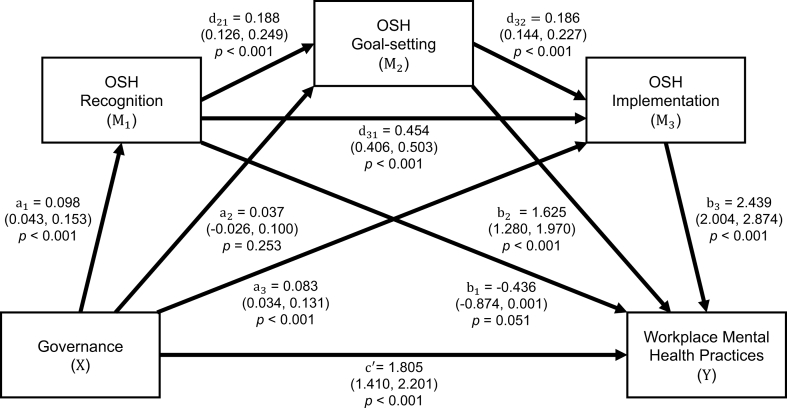
Serial mediation model linking corporate governance to workplace mental health practices through structured occupational safety and health (OSH) engagement.

Fig. 2 illustrates the serial mediation model linking corporate governance, OSH engagement stages, and workplace mental health practices, further supporting the second aim. Path analysis confirmed the sequential process. Governance was significantly associated with OSH recognition (a_1_ = 0.10, *p* < 0.001), which in turn predicted goal-setting (d_21_ = 0.19, *p* < 0.001). Goal-setting further predicted implementation (d_32_ = 0.19, *p* < 0.001), and recognition also had a direct influence on implementation (d_31_ = 0.45, *p* < 0.001). Among the three OSH stages, implementation showed the strongest direct association with workplace mental health practices (b_3_ = 2.44, *p* < 0.001), followed by goal-setting (b_2_ = 1.62, *p* < 0.001). Recognition showed a non-significant association (b_1_ = –0.44, *p* = 0.051).

Subgroup analyses by reporting regime supported the robustness of the primary findings across both association and mediation models (Supplementary Section 6). In GLMMs, corporate governance remained significantly associated with mental health practice adoption in both mandatory (IRR = 1.10) and voluntary (IRR = 1.31) reporting groups (*p* < 0.05). Temporal trends and the positive effects of OSH goal-setting and implementation were consistent across subgroups, while OSH recognition remained non-significant. Market effects differed: listing on the Taiwan Stock Exchange predicted higher adoption only in the voluntary group (IRR = 1.52 vs. 1.05).

In mediation models (Supplementary Section 7), the total indirect effect of governance via OSH engagement was significant in both groups, with OSH implementation (M_3_) as the dominant mediator. Mediation explained a larger share of the governance effect in the voluntary group (40%) than in the mandatory group (14%). Notably, the indirect effects through goal-setting (path 2) and the sequential pathway from goal-setting to implementation (path 6) were significant only under voluntary reporting, suggesting stronger internal engagement mechanisms when external mandates are absent.

## 4 Discussion

Stronger corporate governance was positively associated with the adoption of mental health practices. Of the total effect, 83% was direct, while 17% was explained by indirect pathways through structured OSH engagement: recognition, goal-setting, and implementation. Among these, OSH implementation accounted for the largest share of the indirect effect. In contrast, OSH recognition and goal-setting alone showed no direct association, suggesting that early-stage recognition, without structured follow-up engagement, may not translate into significant change.

Corporate governance exerted both direct and indirect effects on mental health practices, with the direct effect reaching 1.81 in the mediation model. These findings reinforce governance as a primary driver of workplace mental health practices. At the strategic level, governance can shape organizational culture by embedding mental health into human capital strategies, signaling leadership commitment, and legitimizing psychosocial risks as core business concerns [15,25]. Empirical findings from Japan's Health and Productivity Management initiative support this perspective. Takahashi et al. (2021) showed that factors such as written health policies, executive-level agenda-setting, and manager education were significantly associated with improved knowledge of health metrics and higher program participation [40]. Beyond strategic influence, governance also enables the institutionalization of engagement processes, embedding mental health priorities into OSH systems and operational routines [15]. Top-ranked companies typically have stronger administrative capacity and disclosure systems, which may facilitate OSH and mental health practices. As mental health becomes increasingly central in ESG reporting, governance plays a critical role in sustaining engagement by embedding priorities into formal structures and reinforcing organizational commitment over time.

Structured OSH engagement constitutes a sequential mechanism through which governance commitments are translated into workplace mental health practices. This staged approach aligns with organizational-level intervention frameworks, which emphasize preparation, risk assessment, action planning, implementation, and evaluation [41]. Recognition represents the first step, indicating whether an organization explicitly identifies mental health and psychosocial risks as part of its OSH priorities [15,34]. In this study, OSH recognition alone was not significantly associated with mental health practice adoption (IRR = 1.00). While initial recognition of OSH responsibilities is essential, it must be followed by concrete planning and implementation to produce meaningful change [41]. Otherwise, recognition may reflect symbolic compliance, raising expectations and increasing administrative burdens without improving support for employees. Previous research has documented similar patterns, where increased governance demands, such as ESG rankings or board-level oversight, have inadvertently elevated stress levels due to compliance pressures [42]. These findings highlight the importance of progressing beyond recognition to goal-setting and implementation, ensuring governance-driven engagement leads to measurable improvements in employee mental health.

OSH goal-setting functioned as a transitional bridge between OSH recognition and OSH implementation. Although it did not emerge as a significant standalone mediator of the relationship between corporate governance and mental health practices, it played a key mediating role in sequential pathways that originated from OSH recognition. Our analysis revealed that goal-setting was independently associated with greater adoption of workplace mental health practices (IRR = 1.17), underscoring its organizational utility despite not being the principal mediator of governance influence. These findings suggest that setting explicit goals may help build internal alignment, facilitate resource planning, and signal leadership intent, all of which have been identified as key enablers of effective cooperation and successful implementation in complex organizational programs [43].

OSH implementation is the final and most impactful stage of engagement, where governance strategies are put into action. Implementation demonstrated a significant association with mental health practice adoption (IRR = 1.14), accounted for the largest indirect effect (0.20, 53%), and appeared in nearly all mediation pathways. It encompassed concrete measures such as OSH training, certified systems, wellness programs, and reporting of injury or illness outcomes [14,15,[35], [36], [37]]. These findings suggest that OSH implementation contributes to workplace mental health by translating governance priorities into recurring practices that embed mental health into daily organizational routines [2,15,41,44].

This study has some limitations. First, it relied on publicly disclosed OSH and mental health information, which may not fully reflect actual implementation. Some reported practices may be symbolic or selectively disclosed. In contrast, companies with limited disclosure may still engage in meaningful but unreported practices. Reported practices may also differ from employees' lived experiences. Future work incorporating organizational culture could clarify how governance translates into practice. Second, although longitudinal data were used, the observational design limits causal inference. The serial mediation model offers a theory-driven framework to explore pathways, but cannot confirm directionality. Third, the focus on Taiwanese-listed companies may limit generalizability to other sectors or countries with different regulatory and disclosure environments. Cross-national studies could help clarify how institutional contexts influence the governance-OSH engagement-mental health relationship. Fourth, the study examined organizational-level practices. Lacking employee-level outcomes prevented linkage between governance and direct health or behavioral outcomes, thereby limiting the ability to capture employees' lived experiences. Future research using employee-level data across more companies could address this gap. Finally, potential differences between mandatory and voluntary companies should be considered when interpreting governance effects. Subgroup analyses showed that findings were robust across reporting regimes, although some mediation paths appeared only under voluntary disclosure. These exploratory patterns suggest that governance effects are unlikely to be driven solely by symbolic reporting, though some reporting heterogeneity may remain. Future research using stratified or international samples could further examine how the reporting context influences the governance-OSH engagement-mental health pathway.

## 5 Conclusion

Corporate governance plays a central role in advancing workplace mental health practices by influencing how organizations prioritize, resource, and implement psychosocial risk management. Companies with stronger governance adopt more concrete measures—such as counseling services, employee assistance programs, and psychosocial risk evaluation—and part of this effect operates through structured OSH engagement (recognition, goal-setting, and implementation). Implementation emerged as the most consequential stage, showing that governance has the greatest impact when board-level direction is translated into operational systems and routine practices. By clarifying the mechanisms linking governance to mental health practices, this study highlights governance as an important upstream determinant of workplace mental well-being and underscores the need to move beyond symbolic recognition toward actionable implementation.

## CRediT authorship contribution statement

**Ro-Ting Lin:** Writing – review & editing, Writing – original draft, Visualization, Validation, Supervision, Resources, Project administration, Methodology, Investigation, Funding acquisition, Formal analysis, Data curation, Conceptualization. **Lung-Chang Chien:** Writing – review & editing, Writing – original draft, Methodology, Formal analysis. **Chieh-Wen Chang:** Writing – original draft, Formal analysis, Data curation. **Yu-Chi Liao:** Writing – review & editing. **Tomohisa Nagata:** Writing – review & editing, Methodology.

## Informed consent

N/A.

## Ethics statement

N/A.

## Registry and the registration no. of the study/trial

N/A.

## Animal studies

N/A.

## Declaration of generative AI in scientific writing

During the preparation of this work, the authors used Grammarly and ChatGPT to assist with translation and English editing. Final language polishing was performed by a professional editing service (Editage). These tools were employed exclusively for language editing and did not contribute to the generation of scientific content or interpretation of results.

## Funding

This work was supported by the National Science and Technology Council, Taiwan (grant numbers NSTC 113-2221-E-039-016, NSTC 114-2926-I-039-501, and NSTC 114-2314-B-039-061-MY3) and China Medical University, Taiwan (grant numbers CMU112-S-26, CMU113-MF-107, and CMU114-MF-63). The funding source had no role in the study design, data collection, data analysis, data interpretation, writing of the manuscript, or decision to submit the article for publication.

## Conflicts of interest

The authors declare that they have no conflict of interest.
