# Supplementary material for: Corporate Governance and Workplace Mental Health Practices: The Mediating Role of Structured Occupational Safety and Health Engagement
Source: Saf Health Work. 2026 Jan 3;17(1):33–9. doi: 10.1016/j.shaw.2025.12.003 (PMC13023074; doi:10.1016/j.shaw.2025.12.003)
Supplement: Multimedia component 1 [file mmc1.docx]

# **Appendix**

**Table of content**

[**Appendix** 1](#_Toc200627510)

[**Section 1: Governance Score Classification** 2](#_Toc200627511)

[**1.** **Corporate Governance Evaluation System** 2](#_Toc200627512)

[**2.** **Governance Score Operationalization** 2](#_Toc200627513)

[**Section 2: Occupational Safety and Health Engagement Indicators** 3](#_Toc200627514)

[**Section 3: Workplace Mental Health Practice Indicators** 5](#_Toc200627515)

[**Section 4: Company Size and Industry Classification** 7](#_Toc200627516)

[**1.** **Company Size Classification** 7](#_Toc200627517)

[**2.** **Industry Classification** 7](#_Toc200627518)

[**Section 5: Statistical Analysis** 9](#_Toc200627519)

[**Section 6: Subgroup Analyses Based on Generalized Linear Mixed-Effects Models (GLMMs)** 10](#_Toc200627520)

[**Section 7: Subgroup Analyses Based on Serial Mediation Models** 11](#_Toc200627521)

[**References** 12](#_Toc200627522)

# **Section 1: Governance Score Classification**

## **Corporate Governance Evaluation System**

Corporate governance performance was assessed based on Taiwan’s annual evaluation system, conducted by the Securities and Futures Institute under the supervision of the Taiwan Stock Exchange (TWSE) and the Taipei Exchange (TPEx). The evaluation covers four main dimensions: shareholder rights, board structure and functioning, information transparency, and sustainable development [1, 2]. Companies are ranked annually according to their overall performance, with percentile-based classifications ranging from the top 5% to the bottom 100%  [1, 2]. However, disclosure practices varied by year: only the top 20% of companies were publicly disclosed in 2014, and the top 50% in 2015 [1, 2].

## **Governance Score Operationalization**

To ensure comparability across years, governance performance was converted into a binary variable. Companies ranked in the top 5% in any given year were assigned a score of 1 (high governance), while all others were assigned a score of 0 (low governance). This threshold captures only the highest-performing companies in terms of governance and allows consistent analysis over time despite changes in disclosure scope.

# **Section 2:** **Occupational Safety and Health Engagement Indicators**

**Table S1. Definitions of structured occupational safety and health (OSH) engagement indicators**

| **Dimension and indicator** | **Definition** |
| --- | --- |
| 1. **Recognition** |  |
| - 1. OSH Section in Report | The report discloses an OSH-specific section in the table of contents or appendix, with corresponding content in the body text. |
| - 1. Materiality Analysis Process | The report describes the stakeholder engagement and materiality analysis used to identify key sustainability topics. |
| - 1. Occupational Safety in Material Topics | The report discloses occupational safety (e.g., labor safety, industrial safety) as part of the materiality matrix or topic boundary table. |
| - 1. Occupational Safety as Material Topic | The report discloses occupational safety as one of the company’s key material issues. |
| - 1. Occupational Health in Material Topics | The report discloses occupational health (e.g., employee health, labor protection) within the materiality matrix or boundary analysis. |
| - 1. Occupational Health as Material Topic | The report discloses occupational health as a designated material topic. |
| - 1. Mention of ESG or SDGs | The report mentions Environmental, Social, and Governance (ESG) frameworks or the UN Sustainable Development Goals (SDGs), regardless of whether a direct connection to OSH engagement is explained. |
|  |  |
| 1. **Goal-setting** |  |
| - 1. OSH Policy | The report or company website discloses the existence of a formal OSH policy. |
| - 1. Occupational Health in OSH Policy | The OSH policy includes occupational hygiene, occupational health, or employee health. |
| - 1. Occupational Safety Goals | The report discloses specific and measurable goals related to occupational safety. |
| - 1. Occupational Health Goals | The report discloses specific and measurable goals related to occupational health. |
| - 1. Health Certification Goals | The report discloses goals related to obtaining workplace health certifications (e.g., Healthy Workplace Certification). |
| - 1. Comprehensive OSH Plans | The report discloses OSH or employee health protection plans (e.g., maternal protection, overwork prevention, ergonomics). |
|  |  |
| 1. **Implementation** |  |
| - 1. OSH Management System Certification | The report discloses certification under an OSH management system (e.g., ISO 45001, ISO 18001, TOSHMS, CNS 15506). |
| - 1. OSH Committee | The report discloses the existence of an OSH committee or an equivalent structure (e.g., labor-management OSH representation). |
| - 1. Occupational Safety Training | The report describes training activities on accident prevention, safety skills, or hazard awareness. |
| - 1. Occupational Health Training | The report describes programs for health education or employee wellness promotion. |
| - 1. General OSH Implementation | The report describes how OSH is managed or promoted internally, including practices and systems. |
| - 1. Health Promotion Activities | The report describes health promotion activities aimed at physical or mental well-being (e.g., fitness programs, wellness seminars). |
| - 1. OSH Statistics | The report discloses quantitative OSH outcomes (e.g., occupational injuries, disabling incidents). |
| - 1. OSH Awards or Certifications | The report discloses any OSH-related awards or health certifications received during the reporting year. |
| - 1. Occupational Injury/Illness Outcomes | The report discloses qualitative or quantitative outcomes related to occupational injuries or work-related illnesses. Subcontractor data may be included. |
| - 1. Occupational Injury/Illness Statistics | The report discloses quantitative statistics on leave or disability caused by occupational injuries or work-related illnesses. Zero cases are counted. |

Abbreviation: CNS 15506 = Taiwan Occupational safety and health Management System Verification Specification 15506; ISO 45001 = ISO 45001 Occupational Health and Safety Management Systems; ISO 18001 = Occupational Health and Safety Assessment Series 18001; TOSHMS = Taiwan Occupational safety and health Management System.

# **Section 3: Workplace Mental Health Practice Indicators**

**Table S2. Definitions of workplace mental health practice indicators**

| **Dimension and indicator** | **Definition** |
| --- | --- |
| 1. **Planning** |  |
| - 1. Abnormal Workload Disease Prevention Plan | The report mentions plans or measures to prevent diseases related to abnormal workloads. |
| - 1. Workplace Violence Prevention Plan | The report mentions plans or measures to prevent unlawful or violent incidents during work duties. |
|  |  |
| 1. **Measures** |  |
| - 1. Mental Care | The report mentions facilities or resources related to psychological care. |
| - 1. Sports Facilities | The report mentions access to gyms or other physical fitness facilities intended to help relieve employee stress. |
| - 1. Massage Services | The report mentions that massage services are offered to employees to reduce stress. |
| - 1. Employee Travel or Travel Subsidies | The report mentions travel incentives, employee outings, or travel-related subsidies. |
| - 1. Psychological Counseling | The report mentions counseling services, suicide prevention programs, or other psychological support initiatives. |
| - 1. Employee Assistance Program (EAP) | The report mentions the implementation of an employee assistance program. |
| - 1. Working Time Management System | The report describes the presence of a system to manage working hours and explains how it is implemented. |
| - 1. Overwork Prevention Education | The report mentions educational programs aimed at preventing diseases caused by overwork. |
| - 1. Overwork Assessment | The report mentions the use of workload or stress assessments, including questionnaires or screening tools. |
| - 1. Cardiovascular Disease Risk Assessment | The report mentions cardiovascular or cerebrovascular disease risk assessments (e.g., Framingham Risk Score). |
| - 1. Other Overwork Preventive Measures | The report includes any preventive measures for overwork not covered by the aforementioned categories. |
| - 1. Workplace Violence Prevention Education | The report mentions training or awareness programs on preventing and addressing workplace violence. |
| - 1. Gender Equality Education | The report mentions training or awareness programs on gender equality, harassment prevention, or grievance mechanisms. |
| - 1. Monitoring and Alarm Systems | The report mentions the use of monitoring or alarm systems to protect employee safety in the workplace. |
| - 1. Other Workplace Violence Preventive Measures | The report includes any preventive measures for workplace violence covered by the aforementioned categories. |
|  |  |
| 1. **Outcomes** |  |
| - 1. Quantitative Data on Mental Health Support | The report discloses participation rates or other quantitative results related to psychological counseling or EAP services. |
| - 1. Quantitative Data on Overwork Prevention Education | The report discloses data on participation rates or the number of sessions held for overwork prevention education. |
| - 1. Quantitative Data on Overwork Assessment | The report discloses the number of assessments conducted to evaluate overwork risks. |
| - 1. Quantitative Data on Cardiovascular Risk Assessment | The report discloses statistics related to cardiovascular or cerebrovascular risk assessments. |
| - 1. Quantitative Data on Workplace Violence Prevention Education | The report discloses participation or session data on workplace violence prevention training. |
| - 1. Quantitative Data on Gender Equality Education | The report discloses participation or outcome data related to gender equality training. |
| - 1. Mental Health-Related Leave or Disability | The report discloses statistics on employee leave or work-related disability due to mental health issues such as burnout, stress disorders, or occupational psychiatric conditions. Explicit mention of zero cases is also counted. |

# **Section 4: Company Size and Industry Classification**

## **Company Size Classification**

Company size was determined based on the number of employees reported in 2014, which served as the earliest and most consistent baseline across companies. Companies were categorized into two groups:

- Large companies: Those with 1,000 or more employees
- Small companies: Those with fewer than 1,000 employees

This threshold aligns with Taiwan’s regulatory standards for large-scale enterprises and provides a meaningful distinction in terms of organizational resources, operational complexity, and the likely capacity to implement occupational safety, health, and mental health programs.

## **Industry Classification**

Industry classification was based on official industry names obtained from the Market Observation Post System (MOPS) for the 134 listed companies included in this study. These industry labels were cross-referenced with the Statistical Classification of Industries (SIC) (11th revision, 2021) to align with 10 out of the 19 major categories defined by the National Statistics, Republic of China (Taiwan) [3]. To support statistical analysis and enhance relevance to occupational health research, we further consolidated these into three integrated industry sectors.

In addition to administrative classifications, we considered the occupational health burden in each industry by referencing the average number of insurance claims related to cardiovascular and occupational mental disorders from 2014 to 2023, as reported by the Bureau of Labor Insurance, Ministry of Labor [14]. These claims served as proxies for occupational risk exposure and informed the grouping structure, balancing both epidemiological relevance and sample size considerations.

The final industry classification consisted of the following three sectors:

1. Industrial and Manufacturing Sector

This group includes companies engaged in mining, electricity and gas supply, manufacturing (e.g., chemical, semiconductor, biotech, electronics, machinery, and steel), and construction. It aligns with SIC categories B, C, and F. This sector had the highest average number of occupational insurance claims, indicating elevated work-related health risks.

1. Commercial and Service Sector

This sector comprises companies from wholesale and retail trade, transport and storage, hospitality, telecommunications, IT services, and professional or scientific services, corresponding to SIC categories G to J and M. Industries in this group showed moderate levels of occupational disease claims.

1. Finance and Real Estate Sector

This group includes financial institutions, insurance companies, and real estate businesses (SIC categories K and L). It recorded no occupational mental or cardiovascular insurance claims during the observation period and was therefore selected as the reference group in regression models.

Companies that did not clearly fit into these sectors or lacked sufficient representation were grouped based on best fit within this framework. This consolidated classification approach allowed for meaningful comparisons across industry types while ensuring adequate representation and statistical power. A detailed mapping table of industry classifications is provided in **Table S3**.

**Table S3.** Mapping of three-tier industry classification with standardized Statistical Classification of Industries (SIC) by the National Statistics, and industry names from the Market Observation Post System (MOPS), with the number of companies (N)

| Integrated Sector | SIC | MOPS Industry Category |
| --- | --- | --- |
| Industrial and Manufacturing Sector (N=80) | B. Mining and Quarrying (N=1) | - Oil, Gas, and Electricity (N=1) |
|  |  |  |
|  | C. Manufacturing (N=75) | - Automobile (N=2) |
|  |  | - Biotechnology and Medical Care (N=5) |
|  |  | - Cement (N=1) |
|  |  | - Chemical (N=3) |
|  |  | - Computer and Peripheral Equipment (N=12) |
|  |  | - Electric Machinery (N=1) |
|  |  | - Electrical and Cable (N=1) |
|  |  | - Electronic Parts/Components (N=11) |
|  |  | - Food (N=2) |
|  |  | - Iron and Steel (N=3) |
|  |  | - Optoelectronic (N=6) |
|  |  | - Other Electronic (N=3) |
|  |  | - Others (N=1) |
|  |  | - Paper, Pulp (N=1) |
|  |  | - Plastics (N=5) |
|  |  | - Rubber (N=1) |
|  |  | - Semiconductor (N=11) |
|  |  | - Sports and Leisure (N=2) |
|  |  | - Textiles (N=4) |
|  |  |  |
|  | F. Construction (N=4) | - Building Material and Construction (N=3) |
|  |  | - Others (N=1) |
|  |  |  |
| Commercial and Service Sector (N=27) | G. Wholesale and Retail Trade (N=4) | - Electronic Products Distribution (N=1) |
|  |  | - Household (N=1) |
|  |  | - Trading and Consumers’ Goods Industry (N=2) |
|  |  |  |
|  | H. Transportation and Storage (N=8) | - Shipping and Transportation (N=8) |
|  |  |  |
|  | I. Accommodation and Food Service Activities (N=3) | - Tourism and Hospitality (N=3) |
|  |  |  |
|  | J. Information and Communication (N=9) | - Communications and Internet (N=8) |
|  |  | - Information Service (N=1) |
|  |  |  |
|  | M. Professional, Scientific and Technical Activities (N=3) | - Green Energy and Environmental Services (N=1) |
|  |  | - Others (N=2) |
|  |  |  |
| Finance and Real Estate Sector (N=27) | K. Financial and Insurance Activities (N=26) | - Financial and Insurance (N=24) |
|  |  | - Others (N=2) |
|  |  |  |
|  | L. Real Estate Activities (N=1) | - Others (N=1) |

# **Section 5: Statistical Analysis**

We used a multi-step strategy to examine relationships among corporate governance, structured OSH engagement, and workplace mental health practices.

First, grouped weighted quantile sum (GWQS) regression was used to generate stage-specific composite indices for OSH engagement (recognition, goal-setting, implementation). This approach addresses multicollinearity and high dimensionality among the 23 binary indicators, which could otherwise bias model estimates. Each company-year score was derived as a weighted sum of disclosed indicators. The GWQS model was specified as follows:

$$z_{i}=intercept+\beta_{1}\left( \sum_{j=1}^{7} w_{j}v_{ji} \right)+\beta_{2}\left( \sum_{j=1}^{6} w_{j}u_{ji} \right)+\beta_{3}\left( \sum_{j=1}^{10} w_{j}s_{ji} \right)+\varepsilon_{i}$$

where $z_{i}$ represents a workplace mental health practice score; $v_{ji}$, $u_{ji}$, and $s_{ji}$ denote OSH engagement indicators for recognition, goal-setting, and implementation, respectively; and $w_{j}$ (0 ≤ $w_{j}$ ≤ 1) represents the relative weight of each indicator within its group. All weights within each dimension sum to 1. This model was fitted for each year. The resulting weighted indices for each OSH stage over the 10 years were retained as continuous predictors for use in subsequent analyses.

Second, generalized linear mixed-effects models (GLMMs) were used to estimate the longitudinal association between governance, OSH engagement, and mental health practices. The outcome variable was the annual count of disclosed mental health practices across the 10-year period (2014–2023). Independent variables included governance group, three GWQS indices, year, market type, and industry category. Models used the first-order autoregressive covariance structure at the company level. All three GWQS indices were standardized by their interquartile range (IQR) prior to inclusion, allowing interpretation of incidence rate ratios (IRRs) as the expected change in the outcome per one IQR increase in each respective variable. Coefficients were exponentiated to obtain IRRs, representing multiplicative changes in expected counts, with values above 1 indicating increased adoption.

Third, to assess whether OSH engagement mediated the effect of corporate governance, we applied a serial mediation model to reflect the theorized progression of engagement, in which recognition of OSH risks leads to goal-setting, which in turn enables implementation of concrete practices. Governance (high vs. low) was the predictor (X); recognition, goal-setting, and implementation served as sequential mediators (M_1_, M_2_, M_3_); and mental health practice scores were the outcome (Y). The calendar year and its quadratic term were included as covariates. IQR-standardized OSH engagement indices were also used in the mediation models to facilitate effect size comparison. The serial mediation model derived estimated total, direct, and indirect effects of corporate governance on workplace mental health practices, along with bootstrapped 95% confidence intervals based on 5,000 samples. Indirect effects were computed for each possible pathway, including single-stage and chained mediation routes (e.g., X → M_1_ → Y, X → M_1_ → M_3_ → Y, X → M_1_ → M_2_ → M_3_ → Y). An effect was considered statistically significant if the bias-corrected bootstrap confidence interval did not include zero.

To evaluate the contextual consistency of the findings, subgroup analyses were conducted based on reporting requirements. Companies were categorized as either subject to mandatory reporting or voluntary reporting. For each group, subgroup-specific GLMMs and serial mediation models were estimated using the same covariates as the primary analysis. These subgroup analyses served as sensitivity checks to examine whether key associations and mediation pathways held across reporting contexts. All analyses were conducted using R (version 4.3.3) in RStudio (version 2024.12.1, Build 563). Statistical significance was set at p < 0.05.

# **Section 6: Subgroup Analyses Based on Generalized Linear Mixed-Effects Models (GLMMs)**

**Table S4.** Incidence rate ratios (IRRs) and 95% confidence intervals for predictors of mental health practice adoption, stratified by reporting requirement

| **Variable** | **All** | |  | **Mandatory reporting** | |  | **Voluntary reporting** | |
| --- | --- | --- | --- | --- | --- | --- | --- | --- |
|  | **IRR (95% CI)** | **p-value** |  | **IRR (95% CI)** | **p-value** |  | **IRR (95% CI)** | **p-value** |
| Intercept | 2.48 (1.91 to 3.22) | <0.001 |  | 3.10 (2.24 to 4.30) | <0.001 |  | 2.18 (1.39 to 3.43) | <0.001 |
| Year: 2015 vs. 2014 | 1.13 (1.05 to 1.21) | 0.001 |  | 1.14 (1.04 to 1.25) | 0.005 |  | 1.10 (0.99 to 1.23) | 0.084 |
| Year: 2016 vs. 2014 | 1.34 (1.24 to 1.46) | <0.001 |  | 1.37 (1.23 to 1.52) | <0.001 |  | 1.26 (1.08 to 1.47) | 0.003 |
| Year: 2017 vs. 2014 | 1.47 (1.33 to 1.62) | <0.001 |  | 1.50 (1.31 to 1.72) | <0.001 |  | 1.38 (1.18 to 1.61) | <0.001 |
| Year: 2018 vs. 2014 | 1.55 (1.40 to 1.71) | <0.001 |  | 1.62 (1.41 to 1.86) | <0.001 |  | 1.36 (1.18 to 1.57) | <0.001 |
| Year: 2019 vs. 2014 | 1.72 (1.55 to 1.90) | <0.001 |  | 1.81 (1.59 to 2.07) | <0.001 |  | 1.39 (1.16 to 1.67) | <0.001 |
| Year: 2020 vs. 2014 | 1.84 (1.66 to 2.03) | <0.001 |  | 1.88 (1.65 to 2.14) | <0.001 |  | 1.67 (1.39 to 2.01) | <0.001 |
| Year: 2021 vs. 2014 | 1.78 (1.61 to 1.98) | <0.001 |  | 1.82 (1.59 to 2.09) | <0.001 |  | 1.56 (1.30 to 1.86) | <0.001 |
| Year: 2022 vs. 2014 | 1.93 (1.71 to 2.17) | <0.001 |  | 1.97 (1.70 to 2.28) | <0.001 |  | 1.67 (1.36 to 2.06) | <0.001 |
| Year: 2023 vs. 2014 | 2.34 (2.10 to 2.61) | <0.001 |  | 2.40 (2.09 to 2.76) | <0.001 |  | 1.92 (1.51 to 2.45) | <0.001 |
| Governance: High vs. Low | 1.13 (1.06 to 1.20) | <0.001 |  | 1.10 (1.03 to 1.18) | 0.004 |  | 1.31 (1.15 to 1.49) | <0.001 |
| OSH Recognition | 1.00 (0.94 to 1.06) | 0.963 |  | 0.99 (0.92 to 1.05) | 0.675 |  | 1.10 (0.99 to 1.23) | 0.068 |
| OSH Goal-setting | 1.17 (1.12 to 1.22) | <0.001 |  | 1.18 (1.13 to 1.24) | <0.001 |  | 1.14 (1.05 to 1.22) | <0.001 |
| OSH Implementation | 1.14 (1.08 to 1.21) | <0.001 |  | 1.12 (1.05 to 1.19) | <0.001 |  | 1.32 (1.17 to 1.50) | <0.001 |
| Sector: Manufacturing vs. Reference | 0.99 (0.85 to 1.15) | 0.900 |  | 1.02 (0.86 to 1.20) | 0.829 |  | 0.79 (0.59 to 1.05) | 0.103 |
| Sector: Services vs. Reference | 1.01 (0.83 to 1.22) | 0.954 |  | 1.05 (0.86 to 1.27) | 0.632 |  | 0.85 (0.61 to 1.19) | 0.357 |
| Market: TWSE vs. TPEx | 1.33 (1.09 to 1.62) | 0.006 |  | 1.05 (0.81 to 1.36) | 0.693 |  | 1.52 (1.22 to 1.90) | <0.001 |

Abbreviations: CI = confidence interval; IQR = interquartile range; IRR = incidence rate ratio; OSH = occupational safety and health; TWSE = Taiwan Stock Exchange; TPEx = Taipei Exchange. Note: OSH engagement stages (recognition, goal-setting, implementation) were standardized by their IQR prior to analysis. IRRs represent the expected change in the outcome per one IQR increase in each respective variable. The reference group for industry sector is the Finance and Real Estate sector. “Manufacturing” includes industrial and manufacturing companies; “Services” includes commercial and service-related companies.

# **Section 7: Subgroup Analyses Based on Serial Mediation Models**

**Table S5.** Parameter estimates of total, direct, and indirect effects of corporate governance on workplace mental health practice adoptions through OSH engagement, stratified by reporting requirement

| **Effect** | **All** | |  | **Mandatory reporting** | |  | **Voluntary reporting** | |
| --- | --- | --- | --- | --- | --- | --- | --- | --- |
|  | **Estimate** | **(95% CI)** |  | **Estimate** | **(95% CI)** |  | **Estimate** | **(95% CI)** |
| Total effect | 2.19 | (1.75 to 2.62) |  | 2.06 | (1.54 to 2.58) |  | 2.39 | (1.62 to 3.16) |
| Direct effect (not mediated) | 1.81 | (1.41 to 2.20) |  | 1.78 | (1.31 to 2.25) |  | 1.43 | (0.68 to 2.18) |
| Total indirect effect (mediated via OSH engagement) | 0.38 | (0.20 to 0.57) |  | 0.28 | (0.04 to 0.51) |  | 0.96 | (0.60 to 1.36) |
| Indirect effect path 1: via OSH recognition (M_1_) | –0.04 | (–0.10 to 0.00) |  | –0.05 | (–0.12 to 0.01) |  | 0.06 | (–0.13 to 0.25) |
| Indirect effect path 2: via OSH goal-setting (M_2_) | 0.06 | (–0.04 to 0.17) |  | 0.00 | (–0.14 to 0.14) |  | 0.15 | (0.03 to 0.32) |
| Indirect effect path 3: via OSH implementation (M_3_) | 0.20 | (0.08 to 0.34) |  | 0.15 | (0.01 to 0.31) |  | 0.42 | (0.17 to 0.73) |
| Indirect effect path 4: via M_1_ → M_2_ | 0.03 | (0.01 to 0.05) |  | 0.03 | (0.01 to 0.06) |  | 0.05 | (0.01 to 0.10) |
| Indirect effect path 5: via M_1_ → M_3_ | 0.11 | (0.05 to 0.18) |  | 0.13 | (0.05 to 0.22) |  | 0.20 | (0.09 to 0.35) |
| Indirect effect path 6: via M_2_ → M_3_ | 0.02 | (–0.01 to 0.05) |  | 0.00 | (–0.03 to 0.03) |  | 0.07 | (0.02 to 0.14) |
| Indirect effect path 7: via M_1_ → M_2_ → M_3_ | 0.01 | (0.00 to 0.02) |  | 0.01 | (0.00 to 0.01) |  | 0.02 | (0.01 to 0.04) |

Abbreviations: CI = confidence interval; OSH = occupational safety and health.

Note: Based on PROCESS Model 6 with 5,000 bootstrap samples and adjustment for time (linear and quadratic terms), industry, and market status. OSH engagement stages—recognition (M_1_), goal-setting (M_2_), and implementation (M_3_)—were standardized by their interquartile range (IQR). Indirect effects are interpreted per IQR increase in the respective mediator. Effects are considered significant when the 95% confidence interval excludes zero.

# **References**

1. Corporate Governance Center, Taiwan Stock Exchange Corporation, Taiwan. Corporate Governance Evaluation System. [May 17, 2025]. Available from: <https://cgc.twse.com.tw/evaluationCorp/listEn>.

2. Securities and Futures Institute. Corporate Governance Evaluation Results: 1st to 10th Sessions. [March 22, 2025]. Available from: <https://webline.sfi.org.tw/CGE/index_2.asp>.

3. National Statistics, Taiwan. Statistical Classification of Industries (Rev.11 , 2021). 2021 [May 22, 2025]. Available from: <https://eng.stat.gov.tw/News_Content.aspx?n=2597&s=224515>.
